# The weight of Mizaj (temperament) indices in Persian Medicine: A Delphi study

**DOI:** 10.22088/cjim.14.3.513

**Published:** 2023

**Authors:** Morteza Mojahedi, Seyyed Ali Mozaffarpur, Syed Mohd Abbas Zaidi, Hoda Shirafkan

**Affiliations:** 1 Traditional Medicine and History of Medical Sciences Research Center, Health Research Institute, Babol University of Medical Sciences, Babol, Iran; 2 Department of History of Medical Sciences, School of Persian Medicine, Babol University of Medical Sciences, Babol, Iran; 3 Department of Moalajat (Internal Medicine), Hakim Syed Ziaul Hasan Government Unani Medical College, Bhopal, India; 4 Social Determinants of Health Research Center, Health Research Institute, Babol University of Medical Sciences, Babol, Iran

**Keywords:** Qualitative Research, Temperament, Medicine, Traditional, Complementary Therapies, Precision Medicine, Integrative Medicine, Persian Medicine.

## Abstract

**Background:**

Mizaj is an individualized viewpoint in Persian Medicine (PM) that is used for the prevetion of diseases and also treatment. Evaluating Mizaj in the two domains of hotness-coldness, and wetness-dryness, 10 criteria have been introduced, most of them are qualitative. To achieve valid and reliable questionnaires, the weight of these criteria must be determined in assessing the Mizaj.

**Methods:**

In a cross-sectional study with Delphi method, 10 indices were extracted from PM references and sent to PM experts via e-mail. They were asked to score the weight of each index in determining the Mizaj from 0 to 10. The scores ranked and comparing previous preliminary studies, criteria of major and minor were proposed.

**Results:**

Out of 147 invited PM experts, 122 completed the tables. Based on scores, physical functions, physique, and responsiveness of organs obtained the highest scores in the field of hotness-coldness. In wetness-dryness muscle/fat mass and sleep/wakefulness received the highest scores from the viewpoint of experts.

**Conclusion:**

Physical functions, physique (Anthropometry), responsiveness of organs and psychic function can be used as major criteria in Mizaj assessment methods in the hotness-coldness field. In the field of wetness-dryness, muscle/fat mass, sleep/wakefulness, tactile condition and physique (anthropometry) can be considered as major criteria.

A worldwide trend to complementary and traditional medicine recently has been on the rise (1). Different parts of the world also use these medical practices (2). The World Health Organization recommends standardization of diagnosis and treatment in complementary and traditional medicine schools (3). Traditional Persian medicine (PM) also called Unani Medicine, is one of the oldest paradigms of medicine that focuses on lifestyle modification, prevention of diseases, and nature-based treatments. PM is based on the basic principles of the administrative power of the body and a personalized viewpoint (4). Individualized differences in PM are expressed with the concept of Mizaj (5, 6). According to this concept, all human beings are classified based on physical (7), physiological and psychological differences in a range from maximum hotness to maximum coldness (8). They are also classified based on some other characteristics in a range from maximum wetness to maximum dryness (9). This categorization is used to recommend different advice and diets for staying healthy and preventing diseases. To evaluate Mizaj in the two domains of hotness-coldness, and wetness-dryness, 10 criteria have been introduced in PM references (10, 11).

 These include tactile condition (the condition of the skin of the body from the examiner's point of view), muscle/fat mass (the condition of the soft tissue of the body in terms of weight and obesity and also the type of tissue in terms of muscle or fat), hair condition (color, thickness, curl, and stiffness of hair), skin color (various colors of skin), 

physique (anthropometry including ratio of limbs, joints and chest to the whole body etc., pulse and condition of superficial veins), responsiveness of organs (the response of main organs to heat, cold, wet or dry environment or foods), sleep/wakefulness (induction of sleep, duration, and depth of sleep, liveliness after sleep, kind of dreams), physical functions (various physical and physiological functions of the body), body waste (stool, urine, sweat etc.), and psychic function (mental, psychological state and personality of each person) (10). Based on PM references, most of these criteria are qualitative (12, 13) and with uncertain weighting in the final diagnosis. In some of these criteria, studies have been conducted to determine quantitative criteria (8, 11, 14-17). In the study of Mozaffarpur et al., the agreement in determining the Mizaj and also the agreed indices were evaluated by 3 PM experts (16). Also, in the study of Mojahedi et al. as a secondary outcome, the unconscious effects of 10 indices in Mizaj assessment were evaluated (11). However, to achieve valid and reliable questionnaires, the exact weight of each 10 criteria must be determined in assessing the Mizaj (18). Since there is no standard gold standard, the diagnosis of PM experts is considered as the gold standard. In this study, the weight of each of the 10 indices in determining the Mizaj of healthy individuals, in the two domains of hotness-coldness, and wetness-dryness, was assessed.

## Methods

This study is a cross-sectional study that was done from September 2020 to February 2021 at the Research Center of Traditional Medicine and History of Medical Sciences in Babol University of Medical Sciences, Iran. It was approved by the Ethics Committee of Babol University of Medical Sciences. This study was conducted with Delphi method. The Delphi method is a structured process for collecting and classifying the knowledge available to a group of experts (19, 20). 

In this study, based on the main PM references 10 criteria for determining whole body Mizaj were extracted (10). These criteria were set as a table and sent to the PM experts in different universities and regions of Iran. Each physician who graduated in the field of PM in universities was considered expert. At the same time, a letter was sent to them to explain the goals and method of the research. We asked experts to score the weight (or importance) of each index in determining the hotness-coldness of Mizaj as well as the wetness-dryness in a young healthy 20 to 40 years old individual from 0 to 10. A score of 0 meant that the index in terms of clinical experience of the expert, has no role in determining a person's whole body Mizaj, and a score of 10 meant that the index has a very decisive role in determining it. With the decision of research team, the index of physique was divided into 3 indicators including “anthropometry or size of the body”, “pulse” and “superficial veins”. The details of indices are in table1. Correspondence was made via e-mail. Also, telephone calls were made for further explanation and follow-up comments. After collecting the forms, the information was entered into SPSS Version 22 and the mean and standard deviation of the scores were determined. The indices with an average score of less than 5 were considered as “weak”, between 5 to 6 as “moderate”, between 6 to 7 as “good”, and above 7, as an “excellent” score.

## Results

Out of 147 invited PM experts, 122 completed and sent back the tables. The mean age of experts in this survey was 44.33±4.89 years old that 78 (64%) of the participants were females and 44 (36%) were males. The mean duration of their experience in practice of PM was 10.67 ±5.45 years. As a result, physical functions, physique, and responsiveness of organs had the highest scores in the field of hotness-coldness. In wetness-dryness muscle/fat mass and sleep/wakefulness received the highest scores from the experts. The details of scores are shown in table 1. 

**Table 1:** Scores of Mizaj indices from the viewpoints of Persian Medicine experts

Rank of scores	**Hotness-coldness**	Score Mean±SD	**Wetness-dryness**	Score Mean±SD
Excellent	Physical functions Physique (Anthropometry) Responsiveness of organs	7.77±1.85 7.54±2.17 7.47±1.81	Muscle/fat mass Sleep/wakefulness	8.31±1.89 7.40±1.77
Good	Muscle/fat mass Psychic function Tactile condition Physique (Pulse)	6.99±2.62 6.92±1.94 6.82±2.04 6.45±2.26	Physique (Anthropometry) Psychic function Responsiveness of organs Physique (Pulse) Tactile condition Physical functions	6.89±2.80 6.57±2.12 6.27±2.29 6.27±2.55 6.21±2.31 6.18±2.44
Moderate	Skin color Sleep/wakefulness Bodily waste Physique (superficial veins) Hair condition	6.00±1.98 5.86±2.28 5.50±2.33 5.47±2.33 5.20±2.27	Hair condition	5.65±2.27
week	-	-	Bodily waste Physique (superficial veins) Skin color	4.52±2.47 4.11±2.55 3.95±2.55

## Discussion

Based on the results of this study, the indices of physical functions, physique, and responsiveness of organs in hotness-coldness of Mizaj and muscle/fat mass and sleep/wakefulness in the field of wetness-dryness have the most important roles. Although in the references of PM, several indices are used to determine the Mizaj, but their weight and importance in whole body Mizaj assessment is not determined^9^. Giving weight to the proposed indices are essential to develope new valid and reliable questionnaires and other diagnostic tools. In this study, Delphi method as a structured process was used (20). 

In Delphi method, researchers collect and summarize the opinions which have maximum agreement of experts on a particular topic. Collecting and classifying the knowledge of a group of experts is done by distributing questionnaires among them and controlled feedback on the answers and comments received (19). The result of this study was consistent with the results of a study conducted by Mojahedi et al. to measure the effect of 10 indices evaluating Mizaj by PM experts, unconsciously. In that study based on evaluating weighted Kappa (WK), psychic function (WK=0.46), responsiveness of organs (WK=0.44), physical functions (WK=0.41), physique (WK=0.40), muscle/fat mass (WK=0.38), tactile condition (WK=0.37), and skin color (WK=0.37) had the most agreement with the hotness-coldness of the Mizaj. Other indices including body waste, hair condition, and sleep/wakefulness, had no acceptable role in Mizaj evaluation (10). Besides, in a study by Mozaffarpur et al, psychic function, responsiveness of organs, muscle/fat mass, physical functions and tactile condition were mostly used determining hotness-coldness of Mizaj (16). Comparing the outcomes of our result with these two studies, it seems that they are very similar. In the field of wetness-dryness, in Mojahedi’s study, the indices of muscle/fat mass (WK=0.59), tactile condition (WK=0.45) and physique (WK=0.41) had the main role in Mizaj assessment (11). It is supported by the results in Mozaffarpur’s study, that muscle/fat mass, tactile and sleep/wakefulness had the most important role in determining wetness-dryness of the Mizaj (16). These results are accompanied with the result of our study. It seems that muscle/fat mass has the most important role in wetness-dryness of the Mizaj. With Mojahedi’s (11) and Mozaffarpur’s (16) studies, results in proposed major and minor criteria to assess Mizaj. The details are in table 2. The results of our study suggest that, among the 10 indices of Mizaj, hair condition and skin color have the least role in whole body Mizaj assessment, in practice. But we cannot rule out their possible role in assessing the Mizaj of main body organs, including brain and liver. Future studies are required to explore more on this aspect.

**Table 2:** Categorization of Mizaj assessment criteria in Major and Minor criteria

Rank of criteria	**Hotness-coldness**				**Wetness-dryness**			
	criteria	Score Mean±SD	Mojahedi’s study (WK)	Mozaffarpur’s study	criteria	Score Mean(±SD)	Mojahedi’s study (WK)	Mozaffarpur’s study
**Major**	1.Physical functions 2.Physique (Anthropometry) 3.Impressibility speed 5.Psychic function	7.77±1.85 7.54±2.17 7.47±1.81 6.92±1.94	0.41 0.40 0.44 0.46	++ + + +	1.Muscle/fat mass 2.Sleep/wakefulness 3.Tactile condition 4.Physique (Anthropometry)	8.31±1.89 7.40±1.77 6.21±2.31 6.89±2.80	0.59 0.19 0.45 0.41	++ + + -
**Minor**	4.Muscle/fat mass 6.Tactile condition 7.Physique (Pulse) 8.Skin color 9.Sleep/wakefulness 10. Bodily waste 11.Physique (superficial veins) 12.Hair condition	6.99±2.62 6.82±2.04 6.45±2.26 6.00±1.98 5.86±2.28 5.50±2.33 5.47±2.33 5.20±2.27	0.38 0.37 - 0.37 0.21 0.28 - 0.24	++ + - + - - - -	5.Psychic function 6. Responsiveness of organs 7.Physique (Pulse) 8.Physical functions 9.Hair condition 10. Bodily waste 11.Physique (superficial veins) 12.Skin color	6.57±2.12 6.27±2.29 6.27±2.55 6.18±2.44 5.65±2.27 4.52±2.47 4.11±2.55 3.95±2.55	0.34 0.20 - 0.23 0.21 0.31 - 0.25	- - - - - - - -

One of the limitations of this study is the lack of exact guideline to assess 10 Mizaj criteria. Due to the fact that the evaluating method of these indices by different experts may vary, the weight of each of them in determining the Mizaj might also be different.

 Therefore, if unique and standard method for evaluating each of the criteria was introduced, the result would be more conclusive. Although previous studies were conducted on the agreement of experts, it had insufficient sample size (16). 

Furthermore, in the Mojahedi’s study, the weight of these indices was assessed in expert’s diagnosis, unconsciously (11). 

This study is the first one in this field that evaluates the weight of 10 criteria, with alertness of experts and with an acceptable sample size. Large sample size is one of the strengths of this study. Another strength of this study was the use of the Delphi method, which caused participants to express their opinions exactly according to their clinical experiences without being influenced by others. Based on this study, 4 criteria of physical functions, physique (anthropometry), responsiveness of organs and psychic function can be used as major criteria in Mizaj assessment methods in the hotness-coldness field. Moreover, in the field of wetness-dryness, muscle/fat mass, sleep/wakefulness, tactile condition and physique (anthropometry) can be considered as major criteria.
